# Genomics Sheds New Light on the Ancestral Bilaterian Opsin Repertoire and Suggests Rhabdomeric Phototransduction in Xenacoelomorpha

**DOI:** 10.1093/gbe/evaf078

**Published:** 2025-05-26

**Authors:** Samuel Abalde, Ulf Jondelius

**Affiliations:** Department of Zoology, Swedish Museum of Natural History, Stockholm, Sweden; Department of Zoology, Swedish Museum of Natural History, Stockholm, Sweden

**Keywords:** opsins, phototransduction, photoreception, Xenacoelomorpha, Bilateria, Nephrozoa

## Abstract

Animals use opsins, light-sensing, transmembrane proteins, to detect light, which is then converted into an electrical stimulus in the photoreceptors through a process known as phototransduction. The steady generation of genomic data has facilitated the description of the opsin diversity and photoreceptor activity in different animals. However, Xenacoelomorpha still represents an important gap in our understanding of opsin evolution from early animals. Characterized by extreme morphological simplicity, some Xenacoelomorpha present ocelli, eyespots, and even eyes with lenses, but no ciliary or rhabdomeric photoreceptors have been described. Here, we have leveraged all available genomic data from this group to characterize their opsin repertoire and to annotate the genes involved in the two main phototransduction cascades, ciliary and rhabdomeric, in these worms. Only six opsin types were found in Xenacoelomorpha, with no more than three in any given species. Among them, we annotated Anthozoa I, insofar thought to be cnidarian-specific, and another one specific to xenacoelomorphs. Despite their enigmatic position in the animal tree, we extended these findings to other animals, evaluating the two main phylogenetic hypotheses—the Nephrozoa and Xenambulacraria hypotheses. We propose that the opsin repertoire of the ancestral bilaterian was composed of either seven opsins or 11, depending on the phylogenetic hypothesis, and not nine as previously suggested. We argue that the Nephrozoa hypothesis explains opsin evolution more parsimoniously than the alternative Xenambulacraria hypothesis. Furthermore, our data also suggest that despite the lack of the typical rhabdomeric or ciliary photoreceptors, xenacoelomorphs use rhabdomeric phototransduction.

**Significance**

Despite the description of the opsin diversity in many animal groups, the lack of data from key taxa limits our understanding of opsin evolution in Metazoa. One such group is Xenacoelomorpha, whose morphology and genomic content have repeatedly suggested an early divergence within Bilateria. Within Xenacoelomorpha, we annotated opsins thought to be exclusive to cnidarians, as well as xenacoelomorph-specific opsins, which do not fit in the currently accepted hypothesis of opsin evolution. We incorporate these findings to the current state-of-the-art and propose two alternatives (due to the uncertainty in the phylogenetic position of this group) to explain opsin evolution. Furthermore, despite the lack of the typical ciliary and rhabdomeric photoreceptors, our analysis indicates that xenacoelomorphs use a mechanism typical of many other invertebrates, rhabdomeric phototransduction, to convert light into an electric signal.

## Introduction

The capacity to sense and react to light is fundamental for the survival of many animals. Beyond image forming, light guides activities such as regulating the circadian rhythm, phototactic movements, or migration, among many others (Nilsson and Bok 2017). The detection of light occurs within photoreceptors, which in animals can be separated into two categories, ciliary and microvillar (also known as rhabdomeric), with different light-sensing proteins and phototransduction (i.e. the conversion of the light stimulus into an electrical impulse) cascades (Nilsson 2021).

Cnidarians and bilaterians use opsins bound to a chromophore (either 11-cis-retinal or all-trans-retinal) to detect light. Opsins are transmembrane G-protein-coupled receptors with seven transmembrane domains, characterized by the presence of a lysine residue in the seventh helix (termed K296 in reference to the bovine rhodopsin where it was described) (Terakita 2005). Traditionally divided into two (ciliary and rhabdomeric opsins) or three (plus tetraopsins) main groups, ciliary and rhabdomeric opsins were considered characteristic of each photoreceptor type (Nilsson 2021), but systematic survey has led to the discovery of new opsin groups, such as the onycopsins (Hering et al. 2012), insect-specific opsins (Henze and Oakley 2015), or the xenopsins (Ramirez et al. 2016). The most taxonomically sound study to date has classified opsins into nine groups that would be present in the ancestral bilaterian and four in the last common ancestor of cnidarians and bilaterians (Ramirez et al. 2016).

The phototransduction cascade is initiated by opsin activation, which triggers changes in the cell's resting potential. In ciliary photoreceptors, this is mediated by cyclic nucleotide-gated (CNG) channels. The activation of phosphodiesterase 6 breaks down cGMP, closing the CNG channels and hyperpolarizing the cell. Then, arrestin binds the opsin to deactivate it and stops the process (Matulef and Zagotta 2003). In rhabdomeric photoreceptors, the light stimulus opens transient potential receptor (TRPC) and transient receptor potential-like (TRPL) channels, which leads to calcium accumulation and cell depolarization, until a Na/Ca exchanger clears the calcium and restores the photoreceptor sensitivity (Fain et al. 2010). Rhabdomeric and ciliary photoreceptors were originally considered typical of protostomes and deuterostomes, respectively, but both types are present in most animals, sometimes even within the same eye, with different functions (Arendt and Wittbrodt 2001). For instance, while adult *Hydra* uses primarily ciliary phototransduction (Plachetzki et al. 2010), both cascades have been described in reef-building corals and jellyfish larvae (Nordström et al. 2003; Mason et al. 2012). The study of new animals for which neither opsins nor phototransduction has been described is necessary to fully understand the evolution of this system.

Xenacoelomorpha are marine worms (except for two freshwater species) that have adapted to environments ranging from shallow to deep water, including mud burrowing, sand dwelling, and phytal and pelagic species (Jondelius and Jondelius 2019). Three main clades are recognized: Acoela, Nemertodermatida (together forming Acoelomorpha, but see Redmond (2024), and *Xenoturbella*. Xenacoelomorphs are morphologically simple animals, without excretory organs, circulatory system, or through-gut, and with simple musculature and nervous system (Jondelius et al. 2019). Yet, some species have ocelli, a cup-shaped pigment cell with a vacuole that contains platelets (Yamasu 1991), or eyespots, epidermal masses of cells, all of the same type, with pigment granules contained within vacuoles (Lanfranchi 1990). Graff (1882) described eyes with lenses in *Proporus venenosus*. However, these photoreceptors do not include rhabdomeres or cilia, despite their demonstrated photosensitivity (Yamasu 1991; Sprecher et al. 2015). Instead, there are sensory cells in contact with the pigment cell (Yamasu 1991) and some neurons coexpress opsins and TRPC channels (Duruz et al. 2021). The coexpression of opsins and TRPC channels suggests a typical phototransduction cascade happening in these neurons, but it is unclear whether the phototransduction follows a similar biochemical cascade as that of rhabdomeric or ciliary photoreceptors.

Understanding the evolution of animal photoreception requires studying the opsin repertoire in Xenacoelomorpha, a group that was hypothesized to be sister to all other bilaterians (Cannon et al. 2016; Juravel et al. 2023). In this light, the bilaterian opsins proposed by Ramirez et al. (2016) are actually nephrozoan (inherited from the last protostome-deuterostome ancestor), and Xenacoelomorpha is needed to extend this hypothesis to the bilaterian ancestor. An alternative hypothesis places Xenacoelomorpha as the sister group of Ambulacraria, the clade containing echinoderms and hemichordates (Kapli and Telford 2020). In this context, the opsins proposed by Ramirez et al. (2016) as common to “most bilaterians” would represent the opsin repertoire of the ancestral bilaterian. Here, we have leveraged all genomic data available for Xenacoelomorpha (49 transcriptomes and four genomes) to (i) describe the opsin diversity within xenacoelomorphs, (ii) extend these findings to other animals and improve our understanding of opsin evolution in Bilateria, and (iii) characterize the phototransduction cascade in this group.

## Results

### Xenacoelomorph Opsins

**Fig. 1.**
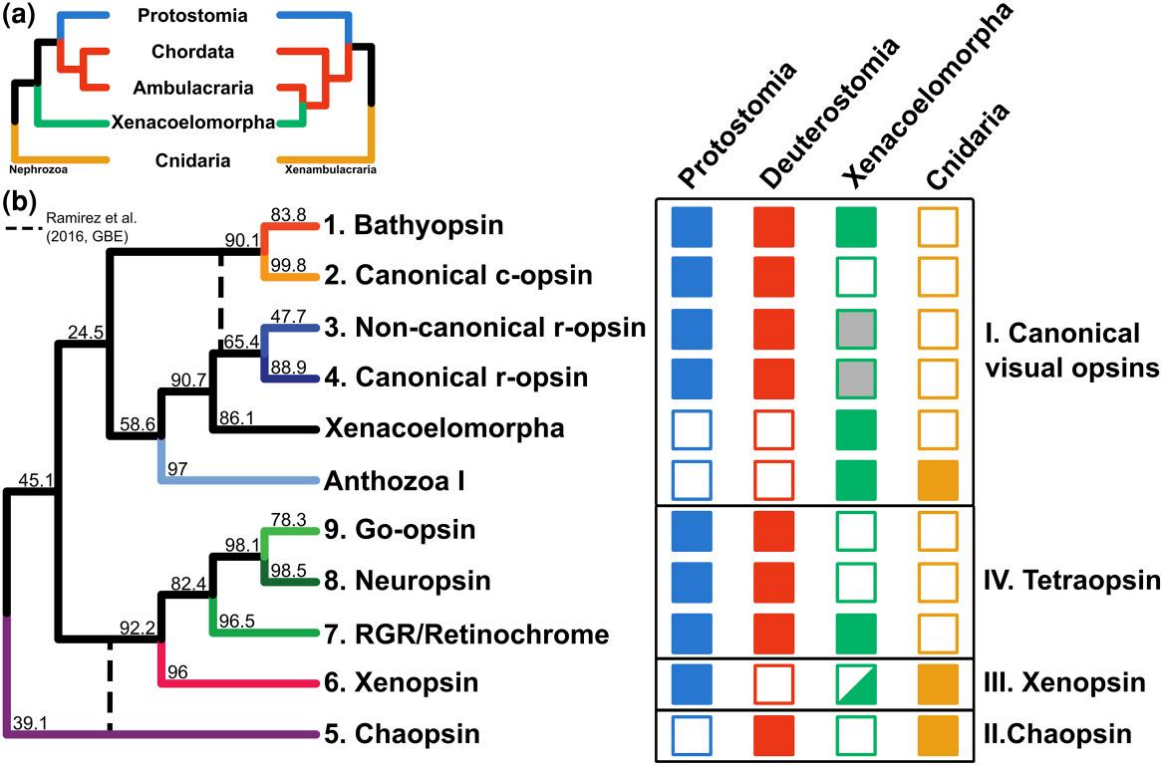
Description of the opsin diversity within main bilaterian clades and cnidarians. a) Summary of the two main competing hypotheses regarding the position of Xenacoelomorpha in the animal tree. b) Presence of opsin families in Cnidaria, Xenacoelomorpha, Deuterostomia, and Protostomia. The opsin presence in Xenacoelomorpha is based on the findings of this study, while opsin distribution in all other clades is based on the results from Ramirez et al. (2016). The cladogram on the left summarizes the phylogenetic relationships among opsin groups recovered by IQ-TREE, and the labels represent the nodal support. Topological differences between this tree and Ramirez et al. (2016) are shown with dashed lines. The tips represent the nine bilaterian opsins proposed by Ramirez et al. (2016), plus “Xenacoelomorpha,” opsins only found in this group. Halfway-colored squares signal opsins without a K296 residue. The black frames group bilaterian opsins derived from the same ancestral opsin, present in the Cnidaria–Bilateria ancestor. Xenacoelomorpha r-opsins form a separate group sister to the canonical and noncanonical r-opsins, indicated by a shade of gray.

Using BLAST searches to annotate putative opsins and phylogenetic trees to filter out false positives, we annotated about 100 opsin sequences from 18 species. These sequences were aligned to the opsins recovered by Ramirez et al. (2016), and a phylogenetic tree was inferred to classify them into opsin families. The phylogenetic tree contained the same opsin groups as in Ramirez et al. (2016), but with one important difference. Noncanonical r-opsins were not monophyletic and their separation from canonical r-opsins received low statistical support. Our tree displayed two topological differences with respect to Ramirez et al. (2016) (supplementary figs. S1 to S3, Supplementary Material online), although low support of some deeper nodes calls for caution. First, chaopsins were recovered as sister to all other opsins, instead of to the xenopsin + tetraopsin clade. Second, the bathyopsins and canonical c-opsins were recovered as a separate clade within the visual opsins (Fig. 1b). All opsins thought to be present in the ancestral eumetazoan were recovered in Xenacoelomorpha, except for chaopsins. Yet, the presence of xenopsins in xenacoelomorphs should be investigated further as none of the two sequences present the conserved K296 residue (Fig. 1b). While the sequence from *Nadina* sp2 was incomplete, the one from *Solenofilomorpha* sp9 was complete and lacked this residue.

**Fig. 2.**
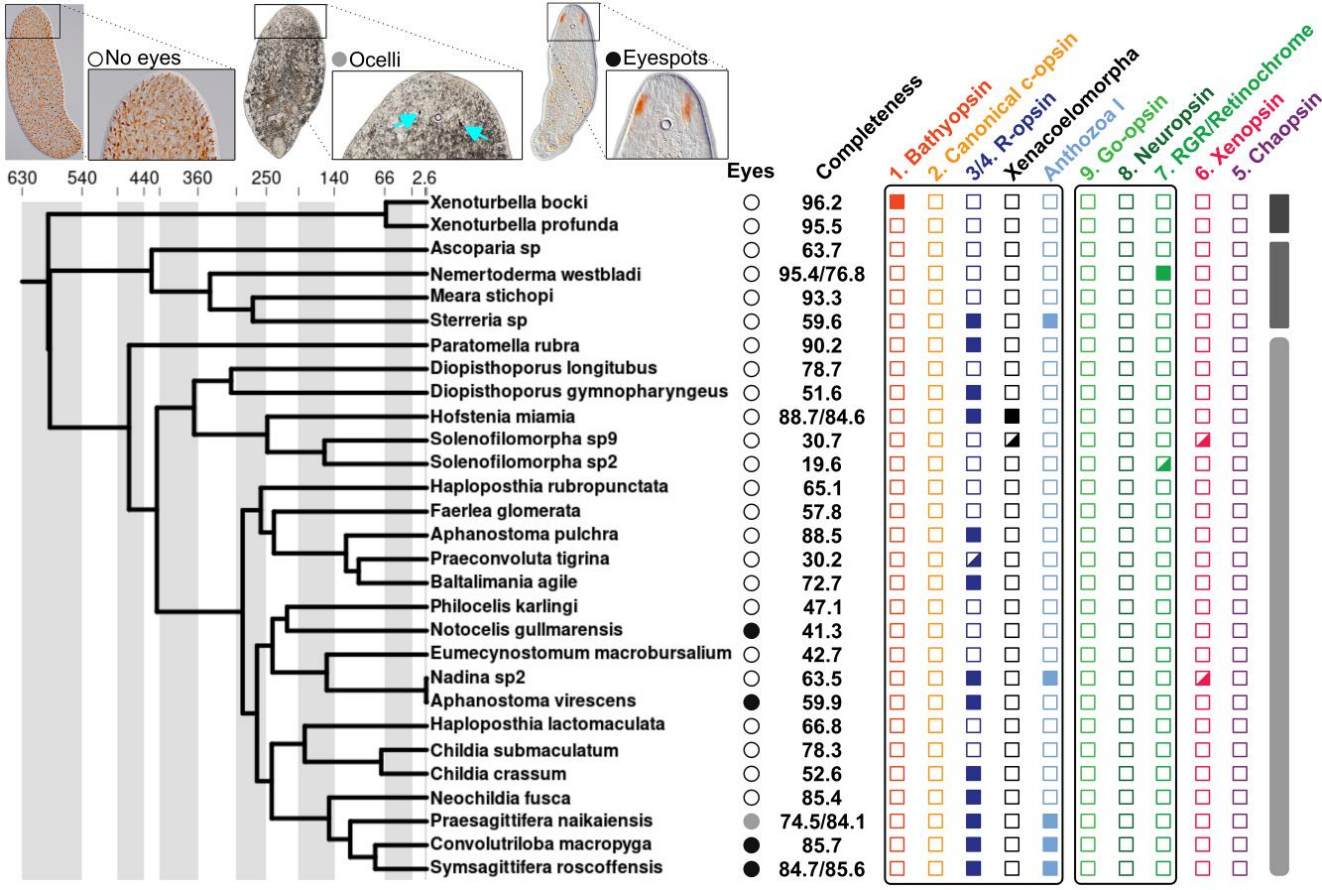
Opsins found in Xenacoelomorpha. The cladogram was drawn after Abalde and Jondelius (2025), using their timetree as reference. Only species with annotated opsins or <60% missing BUSCO genes were included. The presence of eyes is represented with circles: white circle, no eyes (acoel species: *Thalasoanaperus rubellus*); gray, ocelli (*Amphiscolops* sp., marked with cyan arrows); and black, eyespots (*N. gullmarensis*). Photos taken by Ulf Jondelius. When genome and transcriptome were analyzed for the same species, the two completeness stats are presented: genome/transcriptome. The colored squares and black frames represent the same bilaterian opsins included in Fig. 1 and follow the same color scheme, but note that r-opsins are not separated into canonical and noncanonical. Partially colored squares signal opsins without a K296 residue, either attributed to incompleteness (*Solenofilomorpha* sp2, *Nadina* sp2) or absence of lysine (*Solenofilomorpha* sp9, *Praeconvoluta tigrina*). The three gray bars on the right represent the three main xenacoelomorph clades (top to bottom): *Xenoturbella*, Nemertodermatida, and Acoela.

Only about half of the bilaterian opsins could be annotated in Xenacoelomorpha, corresponding to the earliest diverging genes from each clade. The only annotated tetraopsin was the retinochrome, which was annotated in the nemertodermatid *Nemertoderma westbladi* (complete and with a K296 residue) and in the acoel *Solenofilomorpha* sp2 (incomplete, the presence of a K296 residue could not be verified) (Fig. 2). Within the canonical visual opsins, c-opsins were not found. Despite its position in the tree as sister to both canonical and noncanonical r-opsins, one clade of xenacoelomorph's opsins was supported by the reconciliation analysis with Notung as part of the r-opsins, although its relative position hinders its classification into one of these two opsin families. One bathyopsin sequence was also found in *Xenoturbella bocki*, the only opsin annotated in this clade. Additionally, we found two clades of opsins sister to the r-opsins. One formed a clade with Anthozoa I (cnidarian r-opsins) and included sequences from both Acoela and Nemertodermatida, while the other—formed exclusively by three opsins—was in an intermediate position between these and the r-opsins. The xenacoelomorph-specific opsins occurred in two different acoel species, but were annotated in both the genome and transcriptome of *Hofstenia miamia*, strengthening this finding.

### Phototransduction Pathways in Xenacoelomorpha

Using successive BLAST searches against the two phototransduction cascades described in the KEGG database (Kanehisa and Goto 2000) and a conservative approach to gene annotation, we annotated all phototransduction genes related to the vertebrate and fly pathways in the chordate and protostome proteomes, respectively, demonstrating the suitability of our approach for correctly identifying these genes. Only half of the vertebrate phototransduction genes were annotated in the protostomes, while all but five of the fly phototransduction genes were annotated in the chordates.

Within Xenacoelomorpha, the fly phototransduction pathway was the most complete, lacking only four out of the 18 genes (guanine nucleotide-binding protein subunit beta, inactivation no afterpotential D, arrestin 2, and opsin Rh1) (Table 1; supplementary table S1, Supplementary Material online). The same genes, plus the TRPL protein, were not annotated in the chordates. Only one of the genes annotated in Xenacoelomorpha was not found in Acoela (serine/threonine-protein phosphatase PPEF) or Nemertodermatida (GNG13), and up to four in *Xenoturbella* (GNG13, myosin III, protein kinase CK2, and beta-adrenergic receptor kinase). The vertebrate phototransduction was much more incomplete (Table 1; supplementary table S1, Supplementary Material online). Only five genes were annotated in Xenacoelomorpha (calmodulin, rhodopsin, GNB1, guanylate cyclase activator 1, and solute carrier family 24), all present in Acoela but not Nemertodermatida or *Xenoturbella* (supplementary table S1, Supplementary Material online).

**Table 1.** Annotation of the fly and vertebrate phototransduction pathways (according to the KEGG database) in Xenacoelomorpha, Chordata, and Protostomia

Pathway	KEGG reference	Xenacoelomorpha	Chordata	Protostomia	Gene name
Fly	K00910	X	X	X	GRK
Fly	K02183	X	X	X	CALM
Fly	K02677	X	X	X	PRKCA
Fly	K04515	X	X	X	CaMKII
Fly	K04547	X	X	X	GNG13
Fly	K04634	X	X	X	GNAQ
Fly	K04958	X	X	X	IP_3_Rs
Fly	K04967	X	X	X	TRPC4
Fly	K05692	X	X	X	ACTB_G1
Fly	K05858	X	X	X	PLCB
Fly	K07972	…	…	X	GNBh
Fly	K08834	X	X	X	MYO3
Fly	K13802	…	…	X	Rh1
Fly	K13803	X	…	X	TRPL
Fly	K13804	…	…	X	INAD
Fly	K13805	…	…	X	ARR2
Fly	K13806	X	X	X	DAGL
Fly	K13807	X	X	X	PPEF/PPP7C
Vertebrate	K00909	…	X	…	GRK1₇
Vertebrate	K02183	X	X	X	CALM
Vertebrate	K04250	X	X	X	RHO/OPN2
Vertebrate	K04536	X	X	X	GNB1
Vertebrate	K04548	…	X	…	GNGT1
Vertebrate	K04631	…	X	…	GNAT1₂
Vertebrate	K04948	…	X	X	CNGA1
Vertebrate	K04952	…	X	X	CNGB1
Vertebrate	K08328	X	X	X	GUCA1
Vertebrate	K08718	…	X	…	PDE6A
Vertebrate	K12321	…	X	…	GUCY2D_E
Vertebrate	K12322	…	X	X	GUCY2F
Vertebrate	K13749	X	X	…	SLC24A1/NCKX1
Vertebrate	K13756	…	X	…	PDE6B
Vertebrate	K13759	…	X	…	PDE6G
Vertebrate	K13764	…	X	…	RCVRN
Vertebrate	K13765	…	X	X	RGS9
Vertebrate	K19627	…	X	…	SAG

For data on Acoela, Nemertodermatida, and Xenoturbella, see Supplementary Table S1, Supplementary Material online.

Besides the phototransduction cascade, other metabolic processes are needed to fully characterize the cell's response to light. The metabolism of retinol is needed to syntethyse retinal, the chromophore bound to opsins, while the membrane's permeability to calcium controls the cell's resting potential. Within the retinol metabolism, 23 genes were annotated in Xenacoelomorpha. All of them were present in Acoela, whereas only 11 were found in Nemertodermatida and nine in *Xenoturbella* (supplementary table S2, Supplementary Material online). When we overlay these results over the KEGG pathway map, we see that these 23 genes complete all steps of the retinol pathway (supplementary fig. S4, Supplementary Material online). Similarly, 43 out of 101 genes (Acoela: 37, Nemertodermatida: 28, *Xenoturbella*: 19) of the calcium signaling pathway were annotated (supplementary table S3 and supplementary fig. S5, Supplementary Material online).

## Discussion

Here, we used all available genomic data from Xenacoelomorpha to infer the opsin diversity and phototransduction pathway in this group. One important pitfall is the high levels of missing data for many species, but not at higher taxonomic levels. After combining all transcriptomes and genome annotations, the Xenacoelomorpha dataset includes 99.5% of the BUSCO metazoan genes, Acoela 95.6%, Nemertodermatida 97.4%, and *Xenoturbella* 91.5% (supplementary fig. S6, Supplementary Material online). Thus, while the general patterns at high taxonomic levels should not be affected unless xenacoelomorph opsins are characterized by a high interspecific variability, it might be difficult to fully characterize the opsin repertoire of single species. As for the phototransduction cascade, the high completeness of the combined data suggests all the metabolic components involved in this pathway were annotated.

### Opsin Variation Within Xenacoelomorpha

The only opsin found in *Xenoturbella* was the Bathyopsin, which was exclusive to this group. Two opsins constitute the basic opsin repertoire of Acoelomorpha: r-opsins and Anthozoa I, the latter supposed to be cnidarian-specific (Ramirez et al. 2016). Importantly, the r-opsins correspond to neither the canonical nor the noncanonical r-opsins described for other bilaterians, which could be explained by the retention of an ancestral r-opsin or a higher evolutionary rate resulting in highly diverging r-opsins. RGR/retinochrome may also be present in Acoelomorpha, but the only Acoela sequence does not have a K296 residue. Similarly, xenopsins are potentially present in Acoela, but neither of the two sequences identified contain the characteristic K296 residue, and this annotation should be taken cautiously.

Most of Xenacoelomorpha do not have any visual structure, but there are species with simple ocelli, eyespots, and even eyes with lenses (Graff 1882; Lanfranchi 1990; Yamasu 1991). These most likely do not have a common origin but have appeared several times in different species (Fig. 2). The possession of discrete visual organs in some xenacoelomorphs does not entail a higher abundance of opsins in these species. Despite having eyespots, no opsins were annotated in *Notocelis gullmarensis*, albeit with a relatively incomplete transcriptome (58.7% missing genes), while there are species without them, such as *Nadina* or *Sterreria*, with two (potentially three) opsins. A similar pattern has been described in other invertebrates, where opsin diversity does not correlate with eye complexity (De Vivo et al. 2023). Instead, acoels may also possess photosensitive cells other than the ocelli and eyespots, which could explain the diversity of opsins in this group. *Praesigettifera naikaiensis* regains response to light after ocelli ablation even before pigment cell regeneration (Yamasu 1991), and in situ hybridization in *Symsagittifera roscoffensis* has shown coexpression of opsins and transient receptor channels (necessary for phototransduction) in the periphery of the brain, despite this species having two eyespots flanking the statocyst (Duruz et al. 2021). In addition, there is growing evidence of many nonvisual functions of opsins (Menon et al. 2011; Shen et al. 2011; Leung and Montell 2017; Zanini et al. 2018; Leung et al. 2020), but this is yet to be explored in xenacoelomorphs.

### A New Hypothesis of Opsin Evolution in Bilateria

In recent years, the annotation of opsins from multiple phyla has contributed to the description of the opsin repertoire for several clades and the patterns of gene duplication and gene loss that gave rise to the distribution of opsins that we see today (e.g. (Henze and Oakley 2015; Ramirez et al. 2016; De Vivo et al. 2023). Yet, no study to date has characterized the opsin diversity of xenacoelomorphs, which represents an important gap in our understanding of opsin evolution. Although their phylogenetic position in the metazoan tree remains contentious (Fig. 1a), the survey of xenacoelomorph opsins sheds new light on the opsin repertoire of the bilaterian ancestor regardless of the phylogenetic hypothesis.

**Fig. 3.**
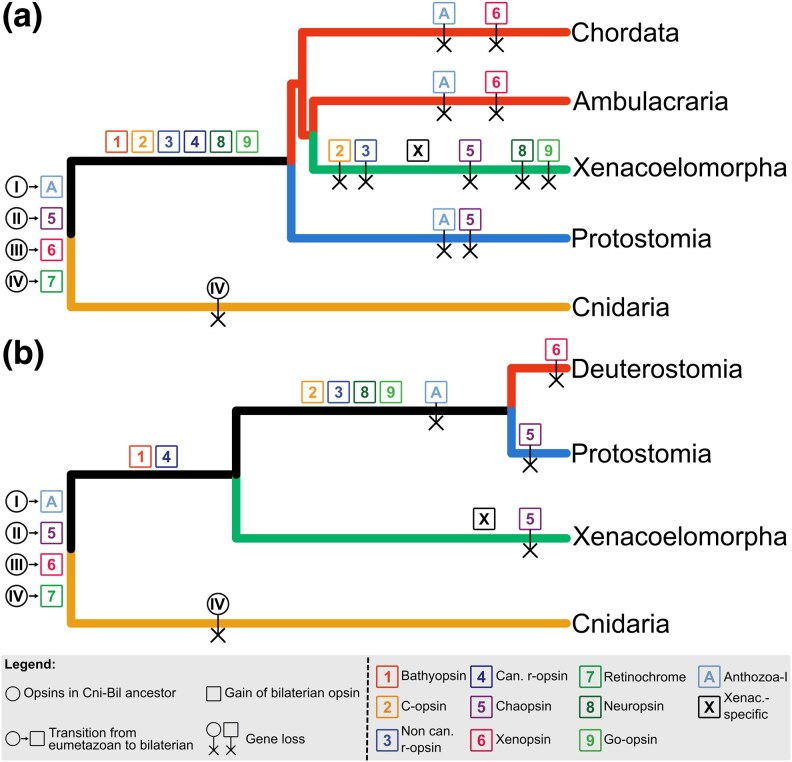
– Patterns of gene gain and loss during opsin evolution under the Xenambulacraria (a) and Nephrozoa (b) hypotheses. Genes are color-coded as in Figs. 1 and 2, but the gene names are also provided in the legend. Due to the impossibility to classify xenacoelomorph's r-opsins into any of the two groups, the canonical r-opsins [4] are used instead. The Roman and Arabic numbers in the circles and squares, respectively, reference the opsins present in the last Cnidaria–Bilateria ancestor and bilaterian opsin families described by Ramirez et al. (2016). The squares named “A” and “X” represent the Anthozoa I and xenacoelomorph-specific opsins, respectively.

Under the Xenambulacraria hypothesis, the bilaterian opsin repertoire proposed by Ramirez et al. (2016) remains almost unchanged, but it should be complemented with Anthozoa I, previously thought to be cnidarian-specific. In this scenario, after the gene duplication from the four eumetazoan opsins, gene loss must have been the main driver of opsin evolution within Bilateria (Fig. 3a). Anthozoa I was lost three times (in Protostomia, Chordata, and Ambulacraria), whereas chaopsins and xenopsins were both lost twice (in Protostomia + Xenacoelomorpha and Chordata + Ambulacraria, respectively). In addition, c-opsins, one of the two r-opsins (assuming a homology between the xenacoelomorph r-opsins and one of the canonical or noncanonical r-opsins), and go-opsins and neuropsins were lost in Xenacoelomorpha.

In contrast, the nephrozoan hypothesis represents a bigger change in the proposed ancestral toolkit, but fewer gene loss and duplication events are needed to explain the current distribution of opsins (Fig. 3b). In this light, the bilaterian toolkit includes six opsins (chaopsins, xenopsins, RGR/retinochrome, Anthozoa I, r-opsins, and bathyopsins), whereas c-opsins, the duplication of r-opsins into canonical and noncanonical, neuropsins, and go-opsins are nephrozoan novelties. Xenacoelomorph-specific opsins seem to be closely related to r-opsins. The two r-opsins might have originated from the xenacoelomorph-specific opsins, which was either lost in the nephrozoan ancestor or it evolved into one of these two families. Here, we assumed a homologous relationship between these and the canonical r-opsins to minimize the number of evolutionary steps. Two subsequent duplications from the ancestral Tetraopsin gave rise to the RGR/retinochrome and the precursor of go-opsins and neuropsins. Only four gene losses, Anthozoa I in Nephrozoa, xenopsin in Deuterostomia, and chaopsin in Protostomia and Xenacoelomorpha, are inferred under the Nephrozoa hypothesis. In contrast to the Xenacoelomorpha hypothesis, in this light opsin evolution might have been driven primarily by gene duplication.

### The Nephrozoa Hypothesis Explains More Parsimoniously Opsin Evolution in Bilateria

Xenacoelomorpha represents one of the most contentious branches in the animal tree. Early phylogenetic analyses recovered Xenacoelomorpha as the sister group of the remaining Bilateria, i.e. supported the Nephrozoa hypothesis (Ruiz-Trillo et al. 1999; Jondelius et al. 2002; Ruiz-Trillo et al. 2002). Phylogenomic analyses based on genomic data have alternately supported the Nephrozoa and the Xenambulacraria hypotheses (Cannon et al. 2016; Philippe et al. 2019; Kapli and Telford 2020; Juravel et al. 2023). Analysis of microRNA (Sempere et al. 2007; Thomson et al. 2014), morphological cladistics (Haszprunar 2016), and gene content (Cook et al. 2004; Jiménez-Guri et al. 2006; De Mendoza and Ruiz-Trillo 2011; Juravel et al. 2023; Abalde et al. 2023) generally support the Nephrozoa hypothesis (but see Schiffer et al. 2024).

The patterns of opsin evolution reported here support the Nephrozoa hypothesis as the most parsimonious, as the opsins annotated in Xenacoelomorpha can be interpreted as an intermediate step between the opsins present in the Cnidaria–Bilateria ancestor and the Bilateria opsins described by Ramirez et al. (2016). Note, however, that in this light said opsins should be considered nephrozoan (present in the last common ancestor of Protostomia and Deuterostomia) and not bilaterian. The Xenambulacraria hypothesis needs 11 gene losses to explain the current patterns of opsin presence, whereas the Nephrozoa hypothesis requires just four (Fig. 3). Furthermore, the Xenambulacraria hypothesis requires several parallel losses of the same genes in distantly related clades: Anthozoa I in Protostomia, Ambulacraria, and Chordata; Xenopsins in Chordata and Ambulacraria; and Chaopsins in Protostomia and Xenacoelomorpha (Fig. 3).

Given the absence of the characteristic K296 residue in the xenacoelomorph xenopsins, we also evaluated the parsimony of the two hypotheses if this opsin was lost in Xenacoelomorpha (supplementary fig. S7, Supplementary Material online). Xenopsins were inferred to be present in the ancestral bilaterian. Hence, the evolution of this one opsin is more parsimonious under the Xenambulacraria hypothesis. The Nephrozoa hypothesis implies parallel loss of xenopsins in Deuterostomia and Xenacoelomorpha, whereas under Xenambulacraria a single gene lost, in the ancestor of Chordata and Xenambulacraria, is required. Yet, the overall pattern of opsin evolution still favors the Nephrozoa hypothesis as the most parsimonious: 10 versus five gene losses, with two parallel losses in Xenambulacraria and only one in Nephrozoa.

### Evidence of Rhabdomeric Phototransduction in Xenacoelomorpha

Studies on cnidarians revealed the presence of both ciliary and rhabdomeric phototransduction in this group (Plachetzki et al. 2010; Mason et al. 2012). The presence of both ciliary and rhabdomeric photoreceptors as early as in the cnidarian–bilaterian ancestor explains their ubiquity within the bilaterians, and both cell types can be present within the same animal (Arendt and Wittbrodt 2001). However, our findings suggest the use of rhabdomeric phototransduction in Xenacoelomorpha and loss of ciliary photoreception in this clade.

None of the central elements of the ciliary phototransduction, such as CNG channels, phosphodiesterase, or arrestin, could be annotated in Xenacoelomorpha, which suggests the loss of this pathway in this group. On the contrary, the use of rhabdomeric phototransduction is supported by the general completeness of this pathway. We annotated several complementary genes that could play an important role during rhabdomeric phototransduction, such as calcium channels and one Na/Ca exchanger (SLC8). The entry of calcium into the cell is essential for TRP activation, while its diffusion through the Na/Ca exchanger restores the photoreceptor sensitivity (Fain et al. 2010). Additionally, we annotated several enzymes that interact with 11-cis-retinal and all-trans-retinal, the two chromophores that bind the bistable r-opsins (Terakita 2005). Rhabdomeric photoreceptors are the main light-sensing structure in protostomes, some cnidarian larvae, and early-diverging deuterostomes (Nordström et al. 2003; Yau and Hardie 2009; Fain et al. 2010). Rhabdomeric phototransduction is more sensitive and can adapt to different light conditions (Fain et al. 2010). These advantages, proposed to explain its expansion within invertebrates, would also apply to xenacoelomorphs.

Although the canonical Rh1 opsin was not annotated within the phototransduction analyses, the presence of r-opsins in our opsin dataset also suggests the use of rhabdomeric photransduction in Xenacoelomorpha. This might be further supported by the coexpression of TRPC4 and *opsin10* described in an in situ hybridization experiment of *S. roscoffensis* (Duruz et al. 2021). *Opsin10* was blast-annotated as Gq-coupled rhodopsin, typical of rhabdomeric photoreceptors, but this annotation should be compared with the opsins annotated in this study to confirm this result. Annotation of Retinochrome in Nemertodermatida and Acoela provides further support for an r-opsin phototransduction cascade in Xenacoelomorpha. Retinochrome isomerizes all-trans-retinal to 11-cis-retinal, the chromophore that forms the rhodopsin (Hara and Hara 1991). The platyhelminthes, a phylum with rhabdomeric photoreceptors, displays a similar opsin configuration, with just r-opsins and retinochrome (De Vivo et al. 2023).

Altogether, we hypothesize rhabdomeric phototransduction is the main light-sensing pathway in xenacoelomorphs, laying the ground for in vivo experiments to further characterize photoreception in this group.

## Materials and Methods

### Data Gathering

All available genomic data of Xenacoelomorpha were included in this analysis: transcriptomes from Abalde and Jondelius (2025), using the same assembly strategy as in that study; the transcriptome of *Xenoturbella bocki* assembled by Brauchle et al. (2018); and the genomes of *Hofstenia miamia* (Gehrke et al. 2019), *P. naikaiensis* (Arimoto et al. 2019), *S. roscoffensis* (Martinez et al. 2023), and *N. westbladi* (Abalde et al. 2023). The chromosome-level genome of *X. bocki* (Schiffer et al. 2024) was published during the late stages of manuscript writing and was not included in the analyses. For the transcriptomes, TransDecoder 5.3.0 (Haas and Papanicolau 2010) was used to extract all open-reading frames with a minimum length of 100 amino acids. The completeness of these proteomes was measured with BUSCO 3.0.2 and the Metazoa_odb9 database (Seppey et al. 2019). In addition, for the phototransduction analyses, we included all annotated, chromosome-level genomes available in GenBank for Protostomia (244) and Chordata (440).

### Opsin Annotation

All reviewed and unreviewed opsins available on UniProt were blasted against the proteomes with diamond 2.0.14 (Buchfink et al. 2021) using the sensitive mode and an e-value of 1e−5. The best-hit proteins in each xenacoelomorph proteome were kept as putative xenacoelomorph opsins. These sequences were blasted against the Uniref90 database (2023_04 release) with the same parameters but allowing three hits per sequence. All sequences for which none of the three hits were opsins were removed. The remaining sequences (including two sequences that blasted only against melanopsins) were aligned to the 765 opsin sequences annotated by Ramirez et al. (2016) and three *Trichoplax* placopsins used as outgroup with MAFFT 7.407 (Katoh and Standley 2013). A phylogenetic tree was inferred with IQ-TREE 1.6.12 (Nguyen et al. 2015) and based on the LG model, using empirical frequencies and a discrete gamma model with four categories. The sequences not recovered within the groups defined by Ramirez et al. (2016) were annotated against the NCBI database in the BLAST webserver and removed if opsins were not among the best hits. The remaining sequences were considered true opsins.

We classified the opsins according to the ancestral opsin repertoire for Eumetazoa and Bilateria proposed by Ramirez et al. (2016), who provided a comprehensive description of opsin diversity, which is complemented with the addition of our data from a missing key taxon. A phylogenetic tree was inferred after aligning the xenacoelomorph opsins to the 765 opsins and three placopsins using the same parameters in MAFFT 7.407 (Katoh and Standley 2013) and IQ-TREE 1.6.12 (Nguyen et al. 2015). After inferring the gene tree, Notung 2.9.1.5 (Darby et al. 2017) was used to reconcile the gene tree and the species tree, using the default duplication and loss weights. Given the contested position of Xenacoelomorpha in the animal tree, we considered both the Nephrozoa (Xenacoelomorpha as sister to protostomes and deuterostomes) and Xenambulacraria (Xenacoelomorpha sister to Ambulacraria, nested within the deuterostomes) hypotheses. Finally, the alignment was manually checked to assess the presence of a lysine residue homologous to the conserved K296 residue traditionally used to differentiate between opsin and nonopsin rhodopsin-type G-coupled receptors (Terakita 2005).

### Annotation of Phototransduction Pathways

Since we were aiming to annotate the presence of these genes in the major clades rather than individual species, the xenacoelomorph proteomes were concatenated into four groups: Xenacoelomorpha, Acoela, Nemertodermatida, and *Xenoturbella*. The protostome and chordate proteomes were concatenated into a Protostomia and a Chordata dataset. The annotation of a gene in one species was sufficient to annotate its presence in the whole group.

To characterize the phototransduction mechanism in xenacoelomorphs, the two pathways described in the KEGG database (‘vertebrate” and “fly’) were taken as reference (Kanehisa and Goto 2000). Retinol metabolism and calcium signaling pathways were also analyzed due to the importance of vitamin A and calcium during phototransduction. Some of the genes included in these pathways are part of major gene families, whose members can have different functions but share a high similarity (and hence are prone to be misannotated). Thus, we focused our efforts on filtering out false positives and dubious annotations. A custom database was created by downloading the genes corresponding to all the UniProt accession numbers associated with each pathway. First, these genes were aligned against the proteomes with diamond 2.0.14 (Buchfink et al. 2021) using the sensitive mode and only the best-hit proteins were retrieved. These proteins were annotated against the full UniProt database using Diamond. All the proteins whose best-hit share an accession number with any of the KEGG proteins were automatically annotated. Then, we extended the annotation to other proteins that could be unequivocally identified by name (e.g. since there are two variants of the adenosin A2, A or B, proteins identified as “Adenosin A2A” were considered, but those simply annotated as “adenosin A2” were not). Finally, the full list of proteins was again blasted against Uniref90, and the annotations were extended to proteins that hit the same Uniref cluster.

## Supplementary Material

evaf078_Supplementary_Data

## Data Availability

All genes annotated in this study have been uploaded to a Figshare repository: https://figshare.com/projects/2024_Xenacoelomorpha_opsins/226836.
