# Supplementary material for: Genomics Sheds New Light on the Ancestral Bilaterian Opsin Repertoire and Suggests Rhabdomeric Phototransduction in Xenacoelomorpha
Source: Genome Biol Evol. 2025 May 26;17(5):evaf078. doi: 10.1093/gbe/evaf078 (PMC12104807; doi:10.1093/gbe/evaf078)
Supplement: evaf078_Supplementary_Data [file SupplementaryFigures.pdf › SupplementaryFigures.pdf]

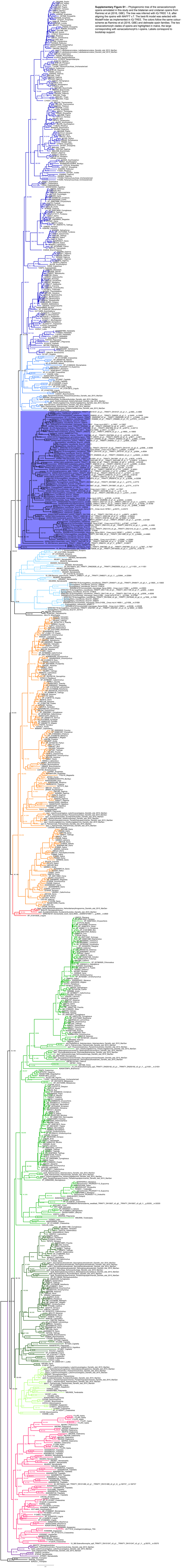

Supplementary Figure S1 – Phylogenomic tree of the xenacoelomorph opsins annotated in this study and the bilaterian and cnidarian opsins from Ramirez et al (2016, GBE). The tree was inferred with IQ-TREE 1.6, after aligning the opsins with MAFFT v.7. The best-fit model was selected with ModelFinder as implemented in IQ-TREE. The colors follow the same colour-scheme as Ramirez et al (2016, GBE) and delineate opsin families. The two xenacoelomorph clades of opsins are highlighted in malve, the large corresponding with xenacoelomorph's r-opsins. Labels correspond to bootstrap support.

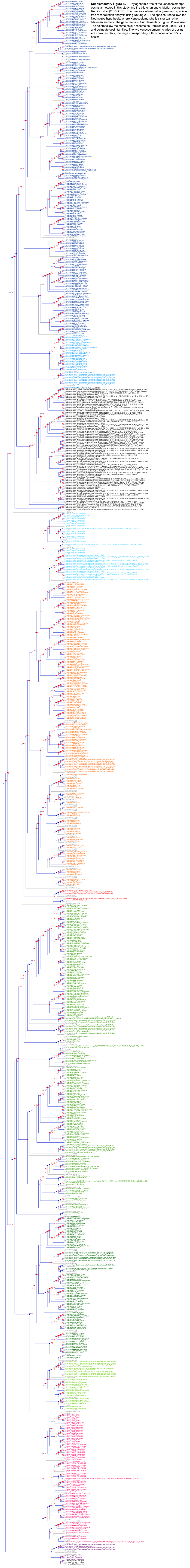

Supplementary Figure S2 – Phylogenomic tree of the xenacoelomorph opsins annotated in this study and the bilaterian and cnidarian opsins from Ramirez et al (2016, GBE). The tree was inferred after gene- and species-tree reconciliation analysis using Notung 2.9. The species-tree follows the Nephrozoa hypothesis, where Xenacoelomorpha is sister toall other bilaterian animals. The genetree from Supplementary Figure S1 was used. The colors follow the same colour-scheme as Ramirez et al (2016, GBE) and delineate opsin families. The two xenacoelomorph clades of opsins are shown in black, the large corresponding with xenacoelomorph's r-opsins.

Image generated with Notung 2.9.1.5, on 12 mar. 2025

Supplementary Figure S3 – Phylogenomic tree of the xenacoelomorph opsins annotated in this study and the bilaterian and cnidarian opsins from Ramirez et al (2016, GBE). The tree was inferred after gene- and species-tree reconciliation analysis using Notung 2.9. The species-tree follows the Xenambulacraria hypothesis, where Xenacoelomorpha is sister to Ambulacraria (Hemichordates + Echinoderms). The genetree from Supplementary Figure S1 was used. The colors follow the same colour-scheme as Ramirez et al (2016, GBE) and delineate opsin families. The two xenacoelomorph clades of opsins are shown in black, the large corresponding with xenacoelomorph's r-opsins.

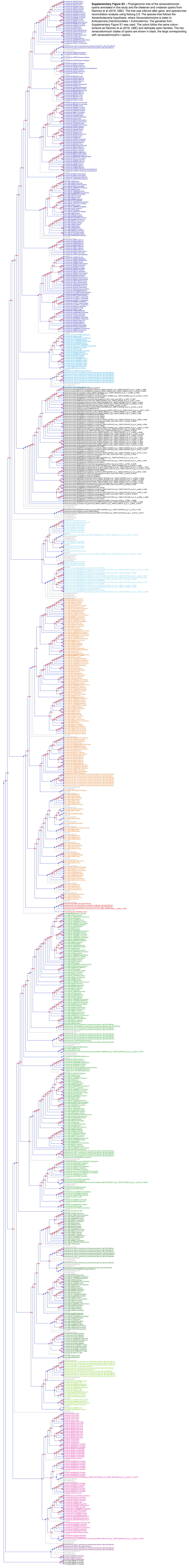

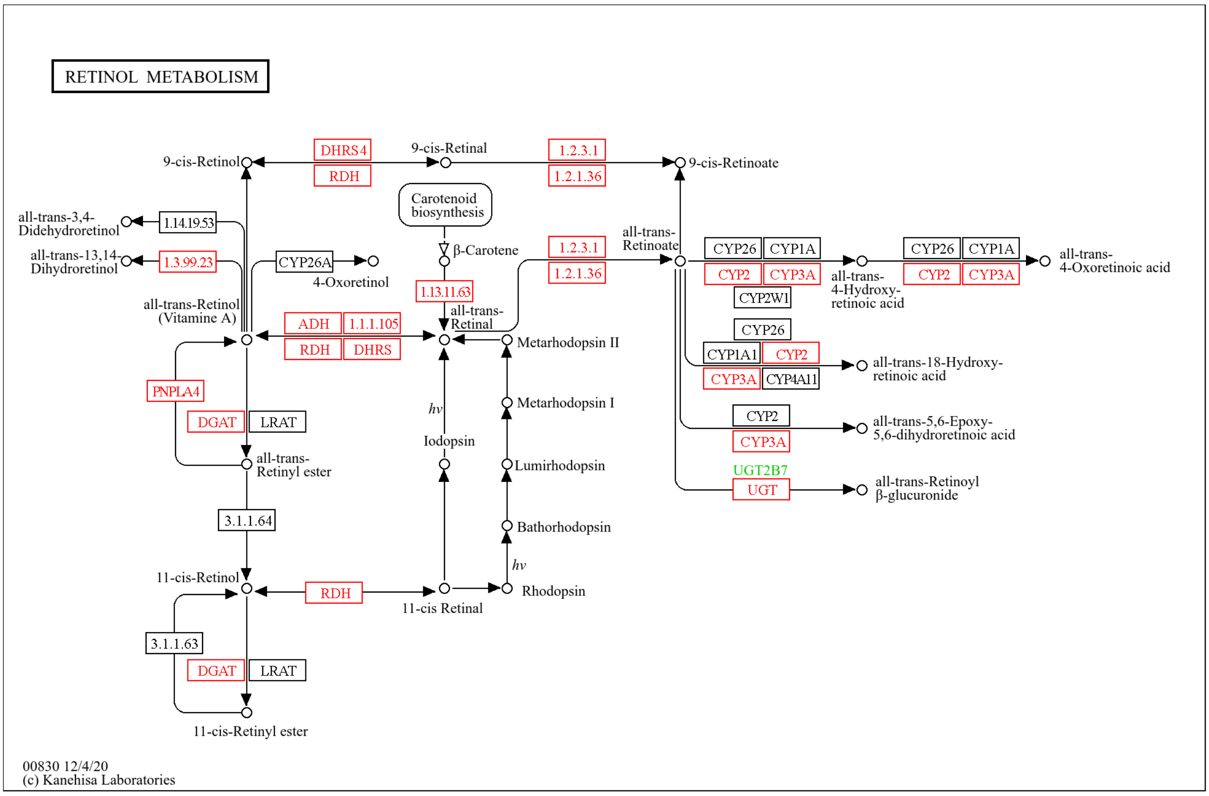

Supplementary Figure S4 - Annotation of the retinol metabolism pathway according to the KEGG database. The genes highlighted in red were annotated in Xenacoelomorpha.

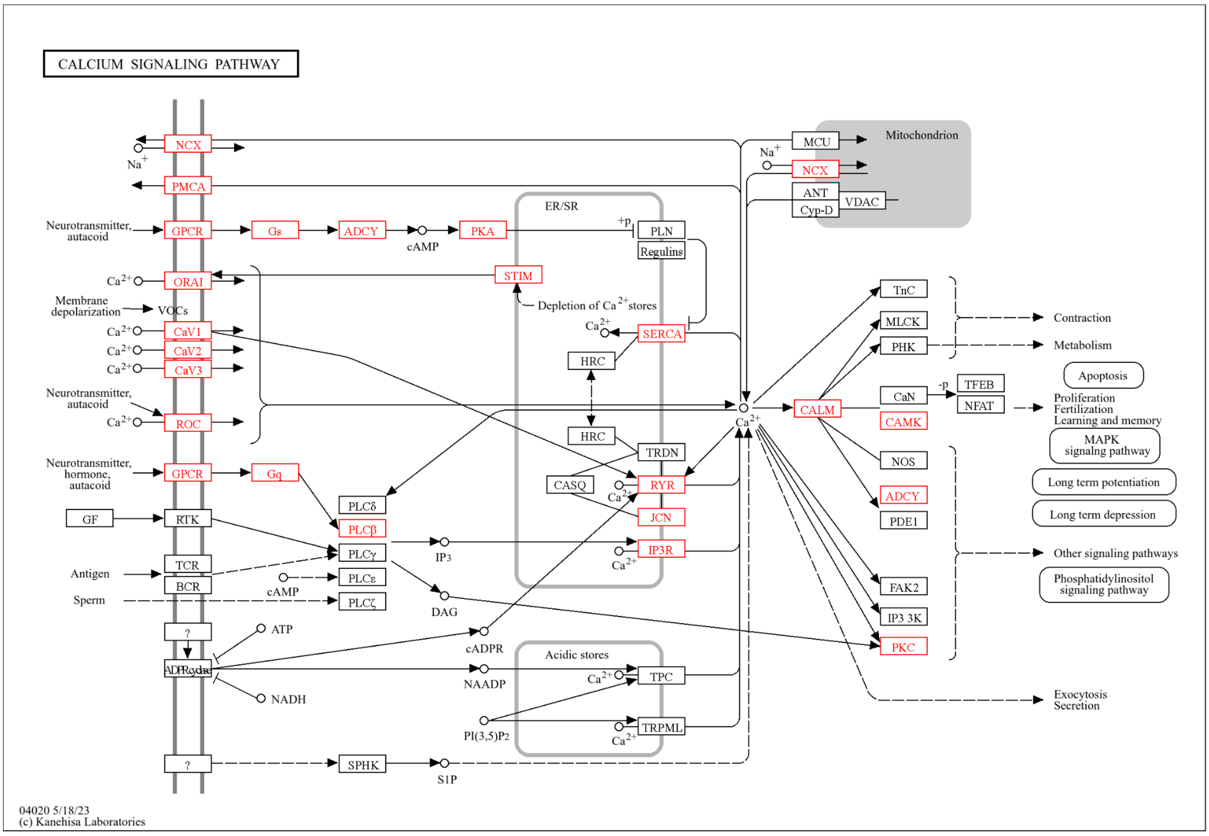

Supplementary Figure S5 - Annotation of the calcium signalling pathway according to the KEGG database. The genes highlighted in red were annotated in Xenacoelomorpha.

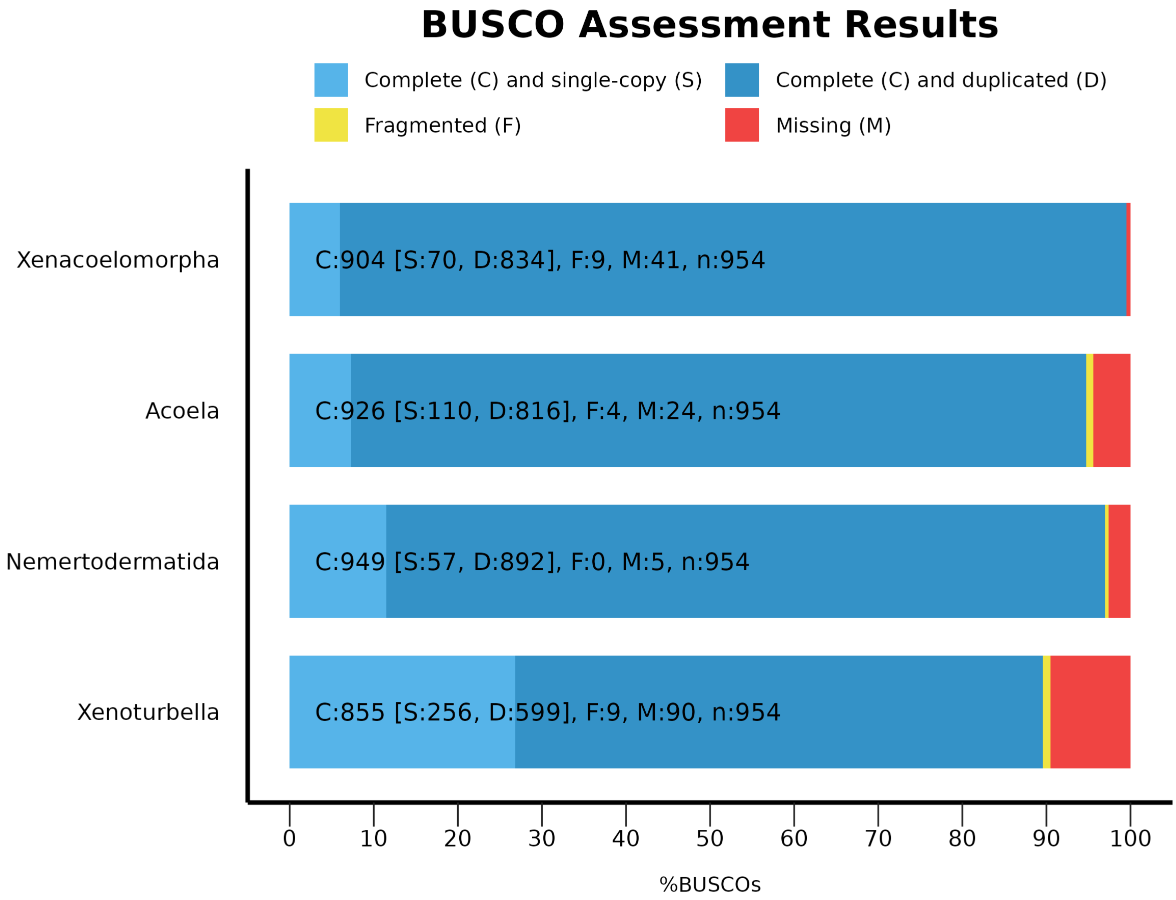

Supplementary Figure S6 – Result of the BUSCO analysis for Xenacoelomorpha, Acoela, Nemertodermatida, and *Xenoturbella*. These datasets were created by combining the genome annotation and the (TransDecoder) inferred protein-coding genes from the transcriptomes. Gene completeness is based on the metazoa_odb10 database.

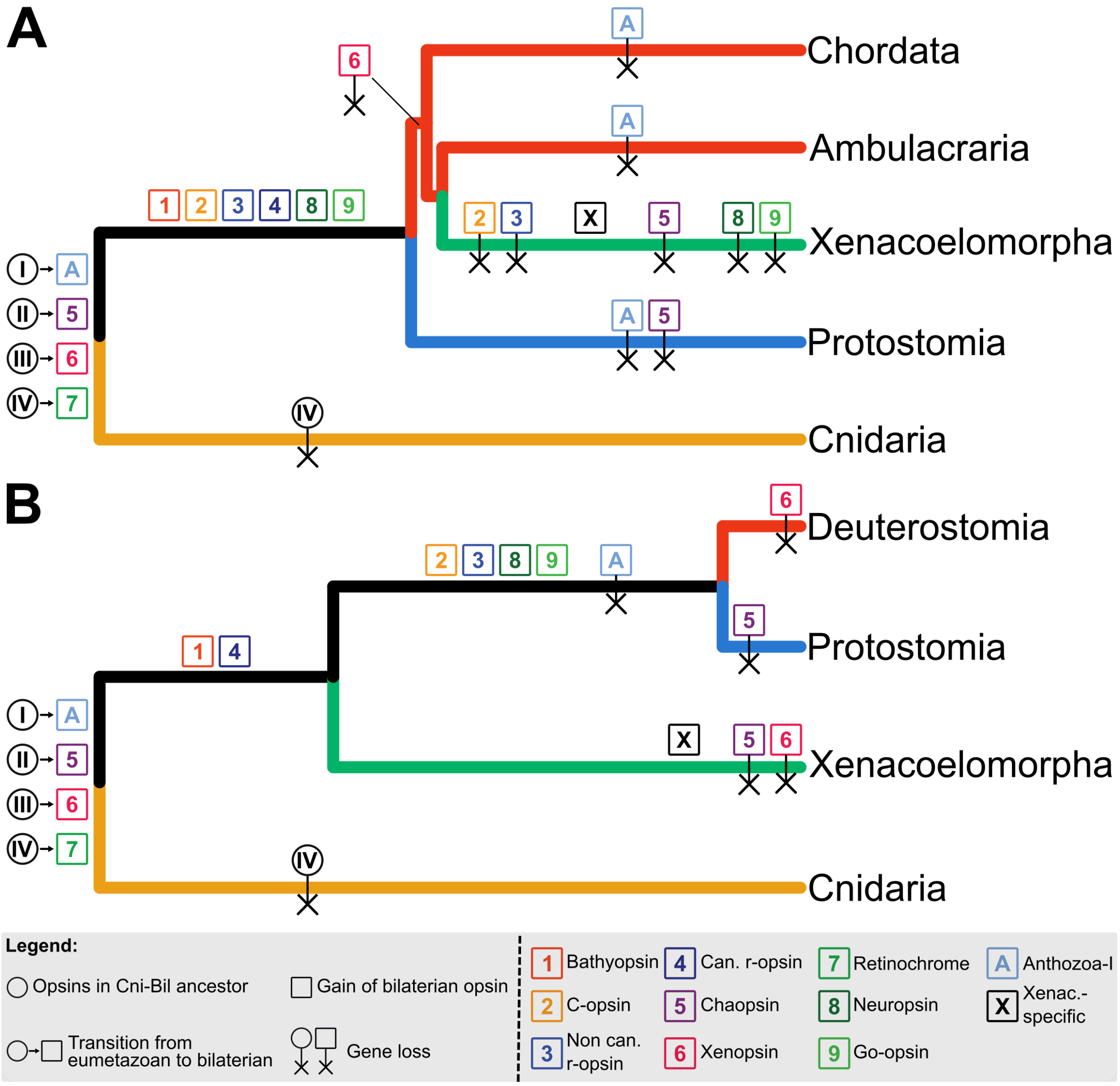

Supplementary Figure S7 – Patterns of gene gain and loss during opsin evolution under the Xenambulacraria (A) and Nephrozoa (B) hypotheses. The main difference with respect to Figure 3 is the assumption that Xenopsins were lost in Xenacoelomorpha, since these sequence do not have the characteristic K296 residue. Genes are colour coded as in Fig. 1 and 2, but the gene names are also provided in the legend. Due to the impossibility to classify xenacoelomorph's r-opsins into any of the two groups, the canonical r-opsins [4] are used instead. The Roman and Arabic numbers in the circles and squares, respectively, reference the opsins present in the last Cnidaria - Bilateria ancestor and bilaterian opsin families described by Ramirez et al. (2016). The squares named "A" and "X" represent the Anthozoa I and xenacoelomorph-specific opsins, respectively.
